# Population-specific responses to pollution exposure suggest local adaptation of invasive red swamp crayfish* Procambarus clarkii* along the Mediterranean French coastline

**DOI:** 10.1007/s11356-024-33775-z

**Published:** 2024-06-13

**Authors:** Marie-Catherine Raffalli, Ana María Bojórquez-Sánchez, Jehan-Hervé Lignot, Diana Martínez-Alarcón

**Affiliations:** UMR-MARBEC, Université de Montpellier, CNRS, Ifremer, IRD, Place Eugène Bataillon, Montpellier, 34095 France

**Keywords:** Invasive species, Pesticides, Local adaptation, Digestive enzymes, Energy use, Genetic diversity, Osmotic pressure

## Abstract

Anthropogenic stressors can have an impact in a broad range of physiological processes and can be a major selective force leading to rapid evolution and local population adaptation. In this study, three populations of the invasive crayfish *Procambarus clarkii* were investigated. They are geographically separated for at least 20 years, and live in different abiotic environments: a freshwater inland lake (Salagou lake) with no major anthropogenic influence and two other coastal wetlands regularly polluted by pesticides along the Mediterranean coast (Camargue region and Bages-Sigean lagoon). Collected adults were genetically characterized using the mitochondrial COI gene and haplotype frequencies were analyzed for genetic variability within and between populations. Results revealed a higher genetic diversity for these invasive populations than any previous report in France, with more than seven different haplotypes in a single population. The contrasting genetic diversity between the Camargue and the other two populations suggest different times and sources of introduction. To identify differences in key physiological responses between these populations, individuals from each population were maintained in controlled conditions. Data on oxygen consumption rates indicate that the Salagou and Bages-Sigean populations possess a high inter-individual variability compared to the Camargue population. The low individual variability of oxygen consumption and low genetic diversity suggest a specific local adaptation for the Camargue population. Population-specific responses were identified when individuals were exposed to a pesticide cocktail containing azoxystrobin and oxadiazon at sublethal concentrations. The Salagou population was the only one with altered hydro-osmotic balance due to pollutant exposure and a change in protease activity in the hepatopancreas. These results revealed different phenotypic responses suggesting local adaptations at the population level.

## Introduction

Invasive species and pollution are among the main drivers of global changes in wetlands (Dudgeon, 2019), which, due to their complex interactions, constitute a “double jeopardy” (Mainka and Howard 2010). Understanding the mechanisms allowing rapid dispersal and adaptive capacities of invasive alien species (IAS) is becoming increasingly essential, given their numbers and impacts on the ecosystems. Among these IAS, invasive crayfish represent a global economic cost of US$ 120 million (Kouba et al. 2022). The red swamp crayfish (*Procambarus clarkii* Girard, 1852) was imported in Europe in 1973 via Spain, where it is now the most abundant among the crayfish species (Gherardi 2006; Loureiro et al. 2015; Dobrzycka-Krahel and Fidalgo 2023). In France, it is the fifth most impactful invasive species, out of 600 species, fauna and flora combined (Souty-Grosset et al. 2016). Its biological characteristics, such as early maturity, rapid growth, large number of offspring, and behavioral flexibility, make it a very successful invasive species. Its tolerance to extreme environments is known: the red swamp crayfish can survive, molt, and breed for days in water with more than 20‰ salinity (Casellato and Masiero 2011; Dörr et al. 2020) and it is regularly found in highly polluted wetlands (Gherardi 2006; Loureiro et al. 2015; Souty-Grosset et al. 2016).

Pollution can impact all four major evolutionary mechanisms (Bickham 2011; Nadeau and Urban 2019): mutations, genetic drift, gene flow, and natural selection. It can lead to an increase in genetic mutation rates, modify gene flow, or act as a bottleneck by greatly decreasing the size of a population. Furthermore, genetic traits that allow better survival or reproduction in a contaminated area can lead to natural selection. The study of evolutionary processes generally considers processes that occur over several hundred of years; but nowadays, the increase in anthropogenic pressures on species and the improvement of high-throughput technology allow for the analysis of rapid evolutionary processes, sometimes over only ten to thirty generations (Klerks et al. 2011; Valladares et al. 2014; Whitehead et al. 2017; Oziolor et al. 2019; Mozdzer et al. 2022). Rapid evolution is observed when a genetic change occurs quickly enough to have a measurable impact on an ecological trait (Hairston et al. 2005; Oziolor et al. 2019; Whitehead et al. 2017). It is common in invasive species and leads to particularly important changes in their invasive capacities (Whitney and Gabler 2008). Rapid evolution may differ within a species: some populations, exposed to different environmental conditions, differ in their physiological responses and genetic structure (Valladares et al. 2014). This is known as local adaptation. Its measurement is based on better physical conditions of each population in its own habitat compared to other populations (Kawecki and Ebert 2004).

This study is focused on monitoring biological markers of physiological state to pesticide exposure of three red swamp crayfish populations living in different salinity and pollution conditions. One population inhabits an artificial inland freshwater lake, the Salagou lake, which is protected from any significant input of pollutants (no agriculture and livestock farming in direct vicinity) (Aquascop 2016). The second population is located in the Camargue region, a protected coastal area (e.g., regulated fishing and surveillance by protection officiers). In this location, some euryhaline shallow wetlands (SNPN, 2020) can regularly reach pollution levels often described as incompatible with the survival of many animal species (CGEDD 2011). From March to September, the Vacarrès lagune in Camargue is located between the “average” and “very poor” categories due to the presence of at least 30 pesticides, azoxystrobin, and oxadiazon included (SNPN, 2020). The third population is located in another coastal area located around the Bages-Sigean lagoon. It faces constant heavy pollution due the presence of at least 35 pesticides and, varying salinities, ranging from brackish up to salinities that can be compared to the Mediterranean Sea (Munaron et al. 2020; PNRNM 2022). Information on the first detection of *P. clarkii* is available by French departments but not for each sampling site in particular. Since 1995, *P. clarkii* has been present in the Bouches du Rhone department (Camargue) and the Hérault department (Salagou Lake), but they appeared in Aude department (Bages-Sigean) until 2001 (Collas et al. 2007; Almerao et al., 2018). To understand the physiological mechanisms that allow this species to inhabit these very diverse environments, in this study, we (i) investigate the genetic diversity of these populations living in different abiotic environments and geographically separated for at least the last 20 years, (ii) determine key physiological and molecular differences between these populations, and (iii) identify population responses when individuals were exposed to a pesticide cocktail of azoxystrobin and oxadiazon. The effects of the pesticide cocktail were assessed by monitoring different biological markers of physiological state. Hemolymph osmotic pressure and oxygen consumption have shown to be good stress indicators in marine invertebrates (e.g., Giesy et al. 1988) and crustaceans in particular (e.g., Hebel et al. 1997; Barbieri 2009; Theuerkauff et al. 2018; Dobrzycka-Krahel and Fidalgo 2023). However, protease and lipase activities have been poorly studied under pollution stress, although their role in digestion and metabolic processes is key for energy production (e.g., Sanchez-Paz et al. 2006; Li et al. 2008; Wang et al. 2013; Martínez-Alarcón et al. 2020). Typically, pesticides target specific metabolic pathways leading to small molecular changes that can potentially induce rapid evolution (Whitehead et al. 2017). The two pesticides considered for this study are among the most frequently found pesticides in water samples from the Vaccarès lagoon in the Camargue area. Azoxystrobin (fungicide) and oxadiazon (herbicide) are both classified at the European level as substances with acute and chronic class 1 toxicity to aquatic environments (EFSA 2010a; 2010b). Since the Salagou population is never exposed to pollutants in their natural habitat, we hypothesize that this population will have a stronger response to the pesticide cocktail in comparison to the Camargue and Bages-Sigean populations.

This study gives insight into the evolutionary and biological processes allowing the successful adaptation of one of the most invasive aquatic crustaceans, and its resistance capacity towards two of the most common pesticides detected in the aquatic habitat of the Camargue area.

## Materials and methods

### Animal sampling and maintenance

**Fig. 1:**
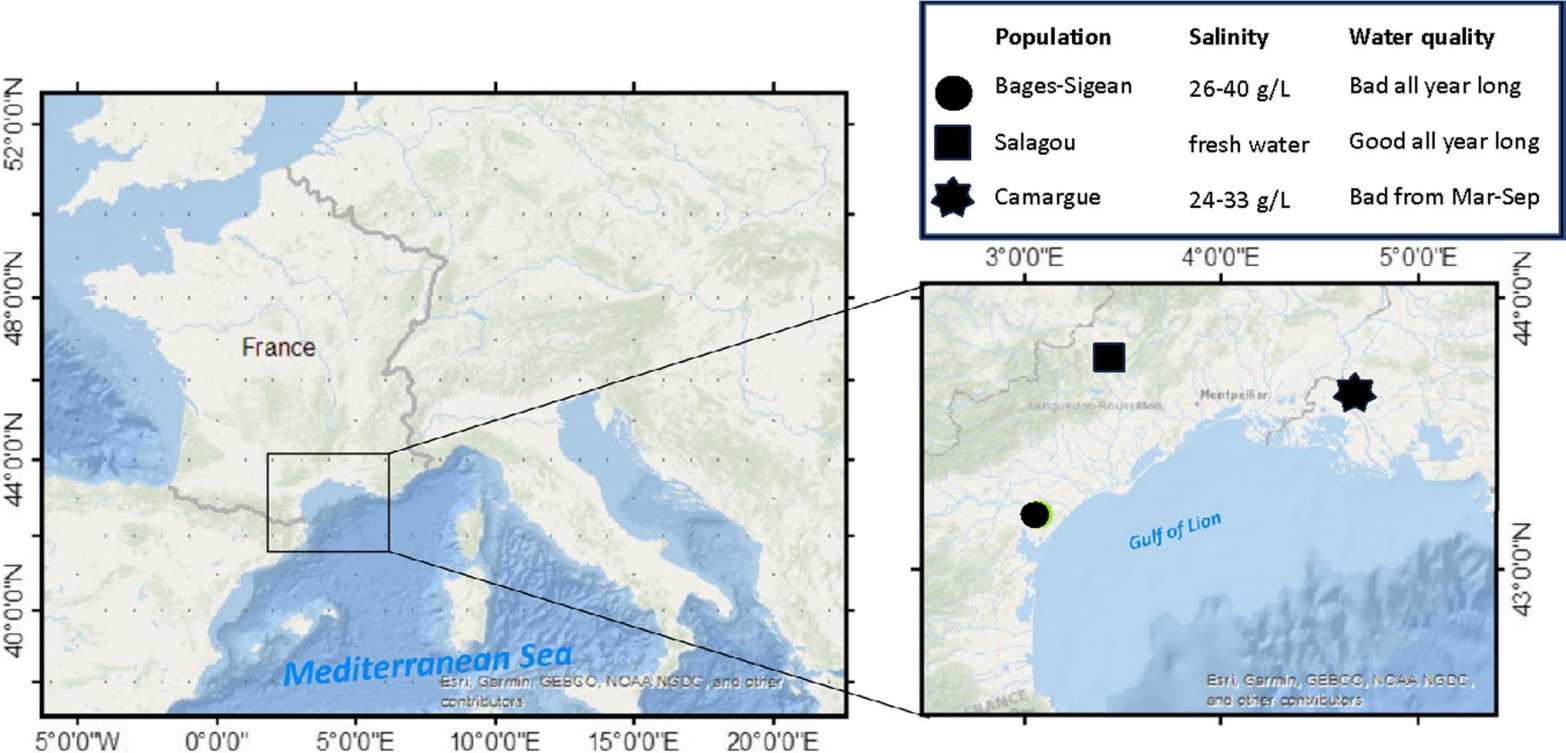
Sampling areas of *Procambarus clarkii* analyzed in this study. The spots on the enlarged map represent the location of the three populations: a star for the Camargue area, a circle for the Bages-Sigean area, and a square for the lentic Salagou Lake. The lake is a 7-km^2^ artificial lake built in 1969. *P. clarkii* individuals are mostly in the waterweed *Lagarosiphon major*, an invasive species introduced in 2009. The coastal study sites are at the interface between freshwater lotic canals and mesohaline lagoons that open to the Mediterranean Sea. The rheology of these water bodies is weak (extremely flat areas) except during coastal storm events and/or flushing of agricultural runoffs

A total of 350 individuals of sizes between 4 and 9 cm were used for this study. Aproximately 150 crayfish of the Camargue population were collected using fixed hoop nets of decreasing mesh size from 13 to 6 mm at the end of the hoop net (“Capechade”), from the Fumemorte canal (43°30′52.6″N 4°40′02.1″E), a drainage canal connected to the Vacarrès lagoon. For the Salagou lake (43°39′45.0″N 3°22′20.5″E), 100 individuals were collected using land nets (30 cm in diameter, mesh size of 6 mm). For Bages-Sigean population, 100 individuals were collected in drainage canals connected to the lagoon (43°07′30.7″N 3°01′20.9″E) using land nets (Fig. 1). Once collected in April and May 2021 and 2022, the three populations were maintained in separate tanks to avoid any mixing before the experiments. Crayfish were kept in large plastic tanks (120 × 80 × 50 cm) filled with recirculated (EHEIM pumps) and filtered dechlorinated tap water kept at 20°C. An equal duration cycle of 12-h light (12L)/12-h dark (12D) was maintained. To avoid predation, inside these tanks, animals were kept in individual transparent, perforated boxes (18 × 12 × 6 cm). Each animal was fed individually two times a week with red blood worms (chironomid larvae).

### Mitochondrial sequence analysis

Total genomic DNA was extracted from muscle tissue with the DNeasy blood and tissue kit (QIAGEN) following the manufacturer’s protocol. COI amplification and sequencing were determined from 23 individuals (Salagou=7, Bages-Sigean=8, Camargue=8). DNA purity and concentration were assessed using the Nanodrop Spectrophotometer (NanoDrop ONE ThermoFisher). DNA was used as a template for polymerase chain reaction (PCR) amplification. All PCRs were carried out in 25-µl reaction volumes in PuReTaq ready-to-go polymerase (Cytiva), 1.25 µl of each forward and reverse primer (10 uM). A fragment of 626 bp corresponding to COI was amplified using universal primers (Folmer et al. 1994) through a PCR program which consisted of 2 min at 95 °C followed by 35 cycles at 95 °C for 1 min, 60 °C for 1 min, 72 °C for 1 min, and a final extension of 5 min at 72 °C. All PCR products were sequenced by Macrogen Europe (Paris, France) using the same primers that were used during amplification.

COI sequences chromatograms were visually inspected for sequencing mistakes. Haplotype network was based on statistical parsimony (Templeton et al. 1992) in the package ‘pegas’ (Paradis 2010) as implemented in the R software version 2022.02.2. Haplotypes were first identified using the function *haplotype* and used to construct a network with the function *haploNet*.

### Experimental design (96-h exposure to a cocktail of pollutants)

#### Acclimation period

Prior to experimentation, individuals were maintained in six glass aquariums (80 × 35 × 45 cm) of 20L for 1 week of acclimatation. In each of the six aquariums (three controls replicates and three experimental replicates), 15 individuals were placed (5 per population). In total, 90 individuals (1/1 sex ratio) were used in each experiment. Each individual was placed in an immersed glass jar, closed by a glass wire net. Water temperature was maintained between 20.0 and 20.4 °C with a 12/12-h photoperiod and 80% of the water was changed daily and any waste was removed. Of all the individuals acclimated previous to experimentation, only one died. This death occurred during acclimatization, before exposure to pollutants, and the individual was replaced. None of the individuals used during this study died during the experimentation.

#### 96-h exposure and choice of pollutant concentration

**Fig. 2:**
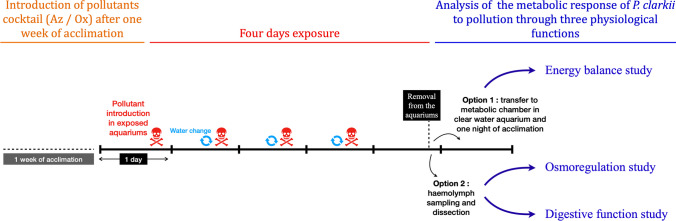
Graphic representation of experimental design. Acclimation time, pollutant exposure time, and analyses performed are indicated. The skull and crossbones symbol represents the introduction of the pollutant cocktail into the aquariums. Blue arrows indicate the time at which 80% of the water was changed

The conditions in which the individuals were kept were identical to those for acclimation but with the addition of oxadiazon and azoxystrobin commonly used for plant protection. This study was based on relevant environmental concentrations of pollutants, using the “EQS-MAC” (maximum acceptable concentration) environmental quality standards as a reference: 0.3 and 0.95 ul/L for oxadiazon and azoxystrobin, respectively (EFSA, 2010). EQS-MAC was used because, in the field, peaks of pollution to azoxystrobin (fungicide) and oxadiazon (herbicide) occur between April and July and not all year round. In this study, we did not assess the LC50 (or EC50) of the used pollutants in *P. clarkii*. However, the used concentrations were below the EC50 for *Daphnia magna* which are 0.53 mg/L and 0.25 mg/L for oxadiazon and azoxystrobin, respectively (Environmental Protection Authority 2024; ECOTOXicology Knowledgebase 2024). Two cocktail concentrations were tested corresponding respectively to 10 and 100 times the MACs. Oxadiazon (Ref: 33382 Sigma-Aldrich, France) and azoxystrobin (Ref: 31697 Sigma-Aldrich, France) were bought in powder form, dissolved in methanol, and added to the experimental aquariums. To maintain a constant concentration of pollutants, 80% of the water was daily renewed, with the corresponding doses of pollutants (Fig. 2); this was based on preliminary test where we saw that the concentration of the pesticides decreased around 10% in 24 h. For the controls, identical volumes of methanol were added in the water, in order to compare only the effect of the cocktail of pollutants on the individuals. For this study, the maximum concentration of methanol used in each aquarium was 0.018 ml/L. Methanol a has high evaporation rate (INRS 2024) and it has been reported that in fish and crustaceans, 0.06 ml/L is the lowest concentration for any observed effect due to methanol during chronic exposure (Kaviraj et al. 2004).

### Indirect flow-through respirometry

The metabolic rate of an organism is an indirect indication of its energy consumption. It gives an idea of the energy expended to maintain vital processes, and can therefore be an indication of an individual’s overall state of health. This can be highly relevant for assessing the effects of exposure to a pollutant. For example, in the pink shrimp *Farfantepenaeus paulensis*, oxygen consumption is inhibited in the presence of cadmium and zinc (Barbieri 2009), and in the green crab *Carcinus maenas*, oxygen consumption is affected by exposure to copper (Hebel et al. 1997). For this analysis, two FireSting O_2_ respirometers (Pyro Science, Germany) were used for parallel measurements in order to evaluate individual oxygen consumption rate. These optical oximeters were connected on one side to a computer with the Pyro Oxygen Logger software and, on the other side, to four optical oxygen sensors (optodes), each of them being connected to the inner side of a metabolic chambers (MC). After 96-h exposure to the azoxystrobin/oxadiazon cocktail, control and exposed individuals were placed in the MC overnight to acclimate (Fig. 2). Respirometry measurements were recorded after acclimatation at T0, T3h, T6h, and T24h. For this purpose, the pumps connected to the metabolic chambers were switched off. Throughout the experiments in the metabolic chambers, an opaque cover was placed over the aquarium to keep the animals in the dark. Therefore, light intensity did not vary during the four sequential measurements. Oxygen concentration values, expressed as micromoles per liter, were directly collected to the PyroScience Logger software every second. The decrease in oxygen saturation was followed for 1 h. Variation of O_2_ consumption per individual corresponds to the difference between the highest and lowest O_2_ consumption values obtained for the four recording periods. Individuals used for respirometry analyses ranged in size from 2.9 to 3.8 cm. Means and standard deviations are summarized in Table 1. The volume capacity of the metabolic chambers was 120 mL. All individuals were therefore able to move, but only slightly. The number of individuals used and the sex ratio are also reported.

**Table 1:** Size (mean ± standard deviation) and number of individuals per population and condition group for respirometry analyses

Population	Salagou		Camargue		Bages-Sigean	
Condition	Control	Polluted 100 MAC	Control	Polluted 100 MAC	Control	Polluted 100 MAC
Size (cm)	3.53 ± 0.31	3.17 ± 0.12	3.27 ± 0.11	3.22 ± 0.11	3.11 ± 0.12	3.19 ± 0.12
Number of individuals	12	16	26	14	29	16
Sex ratio (M/F)	6 / 6	8 / 8	13 / 13	7 / 7	13 / 16	8 / 8

### Hemolymph osmolarity

Maintaining the osmoregulatory function is vital, and the imbalance of this key function during stress can be an indicator of a disturbance in the health of the organism (Hebel et al. 1997; Lignot et al. 2000). Measurements of the hemolymph osmotic pressure (OP) for each individual were carried out after 96 h of exposure, just before individuals were anaesthetised. The hemolymph was taken using a 1-ml hypodermic syringe by inserting the needle through the joint between the Coxa and Basis articles in the right fourth leg. OP was immediately quantified by freezing point depression osmometry (Model 3320, Advanced Instruments, Inc., Norwood, MA, USA). Individuals used for hemolymph osmolarities analyses were in a 1:1 sex ratio when possible, and ranged in size from 5 to 9 cm (Table 2).

**Table 2:** Size (mean ± standard deviation) and number of individuals per population and condition group, for hemolymph osmolarity analyses. *NA* not available

Population	Salagou			Camargue			Bages-Sigean		
Condition	Control	Polluted 10 MAC	Polluted 100 MAC	Control	Polluted 10 MAC	Polluted 100 MAC	Control	Polluted 10 MAC	Polluted 100 MAC
Size (cm)	8.72 ± 0.61	*NA*	8.53 ± 0.69	6.89 ± 0.82	*NA*	6.46 ± 0.72	5.10 ± 0.65	*NA*	5.07 ± 0.92
Number of individuals	29	15	15	29	14	14	23	15	11
Sex ratio (M/F)	15 / 14	8 / 7	8 / 7	14 / 15	7 / 7	7 / 7	11 / 12	9 / 6	5 / 6

### Animal dissections

Individuals were anaesthetised by a low temperature shock (15 min at −20 °C) and then euthanized by the removal of the frontal part of the cephalothorax. The hepatopancreas and muscles of the individuals were dissected, shock-frozen in liquid nitrogen, and stored at −80 °C. All experiments were conducted in accordance with the applicable international, European and national laws applying the principles of replacement, reduction, and refinement (Directive 2010/63/EU of the European Parlament and of the Council of 22 September 2010).

### Digestive enzyme activity in hepatopancreas

#### Individual sizes

Individuals used for the biochemical analyses ranged in size from 5 to 9 cm. Means and standard deviations are summarized in Table 3. The number of individuals used and the sex ratio are also reported.

**Table 3:** Size (mean ± standard deviation), sex ratio, and number of individuals per population and condition group for biochemical analyses

Population	Salagou		Camargue		Bages-Sigean	
Condition	Control	Polluted 100 MAC	Control	Polluted 100 MAC	Control	Polluted 100 MAC
Size (cm)	8.73 ± 0.61	8.53 ± 0.69	6.89 ± 0.82	6.5 ± 0.71	5.01 ± 0.65	5.07 ± 0.92
Number of individuals	10	14	10	15	5	9
Sex ratio (M/F)	6 / 4	7 / 7	4 / 6	6 / 9	2 / 3	4 / 5

#### Extraction and total quantification protein

Samples of hepatopancreas were thawed using the Precellys Keramik-kit (MP, Germany), performing two cycles of 15 s shaking and 20 s pause in between. Thereafter, samples were centrifuged at 15,000*g* for 12 min for proteases analysis and at 10,000*g* for 30 min for lipase analysis at 4 °C. The supernatant, which contained the soluble protein, were aliquoted and stored at −80 °C. Protein quantification was performed after Bradford (1976) with serum bovine albumin as the standard (Sigma-Aldrich).

#### Total protease activity

Total protease activity was determined as the hydrolysis of azocasein, as described by García-Carreño et al. (1994) and modified by Saborowski and Buchholz (1999). The reaction mixture contained 10 µl of the enzymatic extract, 250 µl of 50mM Tris-HCl buffer at pH 8.0, and 250 µl of 0.5% w/v azocasein in 50 mM Tris-HCl buffer at pH 8.0. The reaction mixture was incubated in triplicate for 10 min at room temperature. The reaction was stopped by adding 250 µl of 20% (w/v) trichloroacetic acid (TCA) and cooling on ice for 10 min. The undigested substrate was separated by centrifugation for 5 min at 10,000*g*. The absorbance of the supernatant was then measured in triplicate at 366 nm in a microplate reader. Control assays were done by adding the TCA prior the substrate. Enzyme activity was expressed as the change in absorbance per minute per milligram of protein.

#### Trypsin and chymotrypsin activity

Trypsin and chymotrypsin are among the major digestive endopeptidases of decapods (Tsai et al. 1991; Ma et al. 2005; Vogt 2021). Digestion is a key function, and its disturbance during stress could indicate an energy trade-off (Li et al. 2008; Wang et al. 2013). Trypsin activity was determined as the hydrolysis of Nα-Benzoyl-dl-arginine,4-nitroanilide, hydrochloride (BAPNA: B4875; Sigma-Aldrich), as described by García-Carreño et al. (1994). The reaction mixture contained 50 µl of the enzymatic extract and 250 µl of 0.05% w/v l-BAPNA in 0.1 mM Tris-HCl buffer at pH 7.5. Chymotrypsin activity was determined as the hydrolysis of succinyl-(Ala)2-Pro-Phe-p-nitroanilide (SAPNA). The reaction mixture contained 50 µl of the enzymatic extract and 250 µl of 0.006% w/v SAPNA in 0.1 mM Tris-HCl buffer at pH 7.5. The absorbance was directly measured in triplicates at 405 nm (T0), every 5 min for 30 min in a microplate reader. Specific activities were expressed as the change in absorbance per minute per milligram of protein.

#### Lipase activity

Lipase activity was measured by the hydrolysis rate of *p*-nitrophenyl laurate (*p*NP-laurate) modified after Kordel et al. (1991). The reaction mixture consisted of 0.01 mL of enzyme extract dilution and 190 µl of emulsion. The emulsion was prepared by mixing one part of the substrate solution (10 mm L^−1^ dissolved in DMSO) with 18 parts of solution containing 25 mm L^−1^ Tris, 150 mm L^−1^ and 0.1% (w/v) Triton X-100 (pH 8.0). The enzymatic reaction was carried out at 37 °C and measured over 15 min at 410 nm. One unit of lipase activity is defined as the amount of enzyme releasing 1 µmol of *p*-nitrophenol per minute under the assay conditions.

### Statistical analyses

For the O_2_ consumption rates and the analysis of digestive enzymes, studies were only carried out between control and polluted individuals at 100 MAC. For the hemolymph analysis, the authors studied the effects of 10 and 100 MAC pollution concentrations.

For all statistical analyses, RStudio 2023.12.1 Build 402 software was used. For the O_2_ consumption rates, statistical comparisons between the different populations and conditions were performed using a mixed model with a GLS approach. We used the same model to compare results between the four time periods. For hemolymph osmolalities, normality conditions of the residuals were not met. Therefore, Kruskal and Wallis non-parametric test was used followed by Dunnet post hoc test. For the analysis of digestive enzymes, two-way ANOVAs were used to compare the populations, the conditions, and the interactions between the two factors, followed by Tukey post hoc tests. All the statistical results (tests used, *p*-values, and degrees of freedom) are summarised in Table 4.

**Table 4:** Type of variables, tests used, *p*-values, and total degrees of freedom (Df) for each variable tested (oxygen consumption, hemolymph osmolarity, and enzyme activity)

Measurements (dependent variables):	Oxygen consumption	Hemolymph osmolarity	Enzyme activity			
			Protease	Trypsin	Chymotrypsin	Lipase
Factors (independent variables):	• Population: Salagou, Camargue and Bages-Sigean • Condition: Controls and polluted 100 MAC Repeated measurements over time : times T0, T3h, T6h, and T24h	• Population: Salagou, Camargue and Bages-Sigean • Condition: Controls, polluted 10 MAC and polluted 100 MAC	• Population: Salagou, Camargue, and Bages-Sigean • Condition: Controls and polluted 100 MAC • Interaction between population and condition			
Tests used:	• GLS analysis • Tukey post hoc test	• Kruskal and Wallis non-parametric test • Dunnet post hoc test	• Two-factor ANOVA • Tukey post hoc test			
Significant *p*-values and total degrees of freedom (Df):	T0: Bages-Sigean population (Df = 107): *p-value = 0.049* T3: Bages-Sigean population (Df = 106): *p-value = 0.034* T6: Bages-Sigean population (Df = 107): *p-value = 0.004* T24: Bages-Sigean population (Df = 107): *p-value = 0.061*	Within Salagou population, condition factor (Df = 2): *p-value = 0.008* Polluted 10 MAC–controls (Df = 124): *p-value = 0.037* Polluted 10 MAC–polluted 100 MAC (Df = 83): *p-value = 0.003*	Population (Df = 2): *p-value = 0.019* Salagou–Bages-Sigean (Df = 37): *p-value = 0.014* Salagou controls–Bages-Sigean controls (Df = 14): *p-value = 0.088*	Population (Df = 2): *p-value = 0.026* Salagou–Bages-Sigean (Df = 37): *p-value = 0.019*	Population (Df = 2): *p-value = 8E-6* Bages-Sigean–Camargue (Df = 38) : *p-value = 0.001* Bages-Sigean–Salagou (Df = 37): *p-value = 4E-6* Salagou controls–Camargue controls (Df = 19): *p-value = 0.048* Salagou controls–Bages-Sigean controls (Df = 14): *p-value = 4E-4* Salagou polluted 100 MAC–Bages-Sigean polluted 100 MAC (Df = 12): *p-value = 0.029* Camargue polluted 100 MAC–Bages-Sigean polluted 100 MAC (Df = 13): *p-value = 0.028* Salagou controls–Salagou polluted 100 MAC (Df = 23): *p-value = 0.039*	Controls–polluted 100 MAC (Df = 1 ): *p-value = 4E-4* Camargue controls–Camargue polluted 100 MAC (Df = 24): *p-value = 0.006*

Furthermore, to assess the effect of the individual size on the variables, linear regressions were performed. No linear correlation was found between the variables and the size of the individuals. The *p*-values are summarised in Table 5.

**Table 5:** Results of linear regressions for each variable (i.e., hemolymph osmolarity, enzymes activities, and oxygen consumption): evaluation of the dependence of these variables on the size of the individuals. *p*-values, degrees of freedom, and adjusted R-squared

Variable	Oxygen consumption	Hemolymph osmolarity	Protease activity	Trypsin activity	Chymotrypsin activity	Lipase activity
*p*-value	0.33	0.33	0.40	0.29	0.24	0.56
Degrees of freedom	102	63	45	45	39	39
Adjusted *R*-squared	−0.0006	−0.0003	−0.006	0.002	0.005	−0.016

## Results

### Genetic diversity and haplotype relationships

**Fig. 3:**
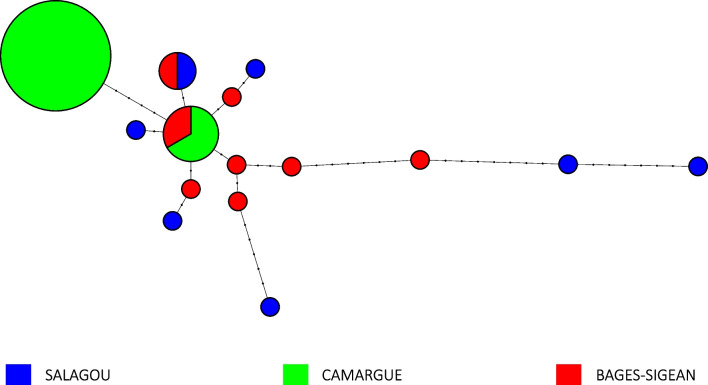
Haplotype network of 15 COI haplotypes from the three studied populations of *P. clarkii*. The circle sizes are proportional to the haplotype frequency within the studied populations. Mutational steps are represented by small black circles. The color of the circles represents haplotypes found in the populations: green for Camargue, red for Bages-Sigean, and blue for Salagou

Among the 23 specimens of P*. clarkii* analysed, a matrix of 626 base pairs of the COI was obtained, yielding 15 haplotypes. In the Salagou and Bages-Sigean areas, at least seven different haplotypes were identified. Only one of the haplotypes is shared between both populations. Genetic diversity is lower in the Camargue population than the other two populations. According to the COI results, only two haplotypes were found in the Camargue (Fig. 3): although 63% of the samples corresponded to the haplotype H2 and 37% to the haplotype H1. This last haplotype was also found in one sample of Bages-Sigean (Fig. 3).

### Oxygen consumption rates

#### Average consumption study

**Fig. 4:**
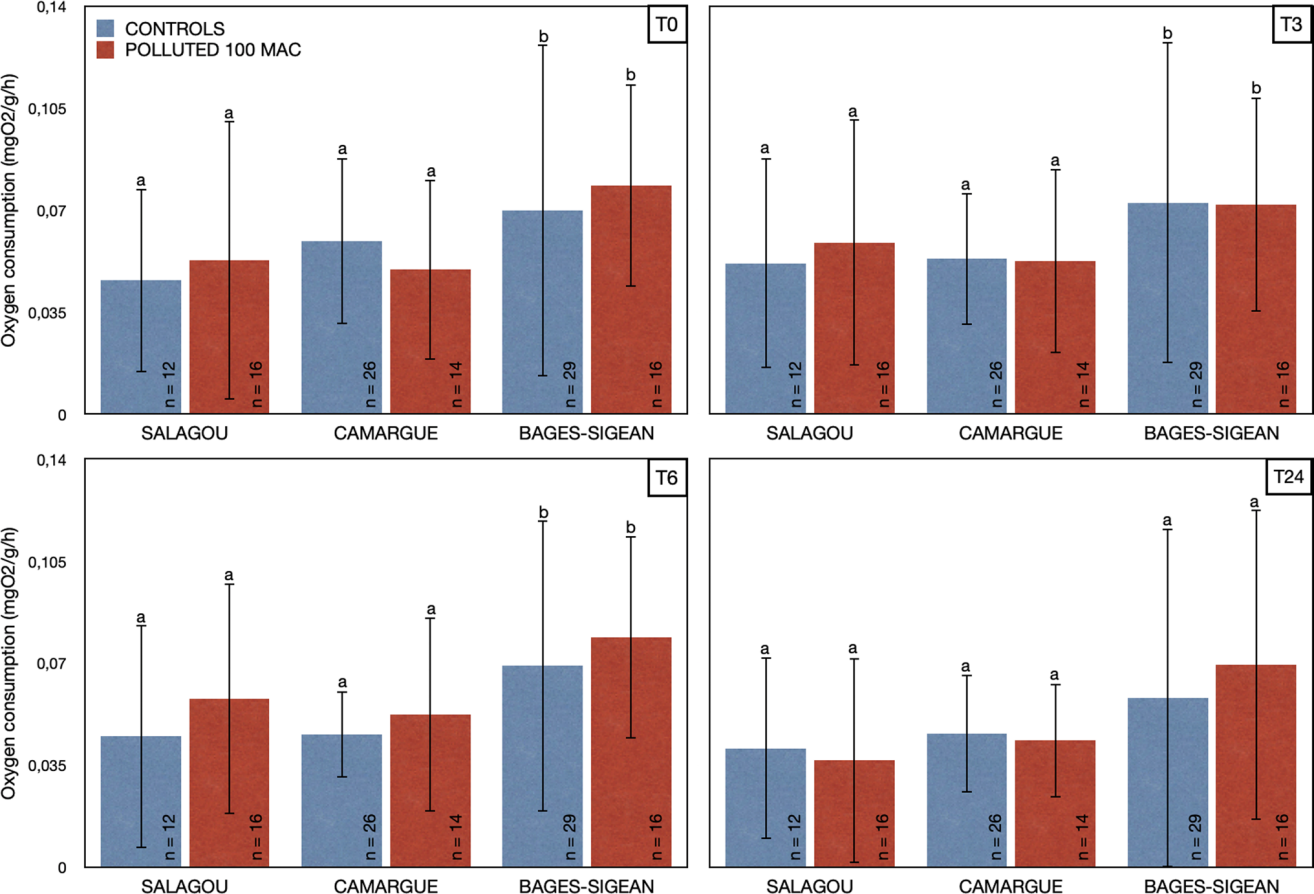
Dissolved oxygen consumption (mgO_2_/g/h) of three populations of *P. clarkii* (Salagou, Camargue or Bages-Sigean) as a function of time (T0, T3h, T6h, and T24h) and exposure to a cocktail of oxadiazon/azoxystrobin pollutants (controls in blue, polluted at 100 MAC in red). Bar plots with means ± standard deviations

The Bages-Sigean population showed a higher O_2_ consumption compared to the other two populations for the first three initial time periods (T0 (*p*-value = 0.049), T3 (*p*-value = 0.034) and T6 (*p*-value = 0.004)), either between controls or between those exposed to a concentration of 100 MAC. For time period T24, the *p*-value between Bages-Sigean population and the other two populations was just above significance level (*p*-value = 0.061). No difference between the different times or between the two conditions (controls and 100 MAC polluted) was found (Fig. 4).

#### Consumption rates as a function of time

**Fig. 5:**
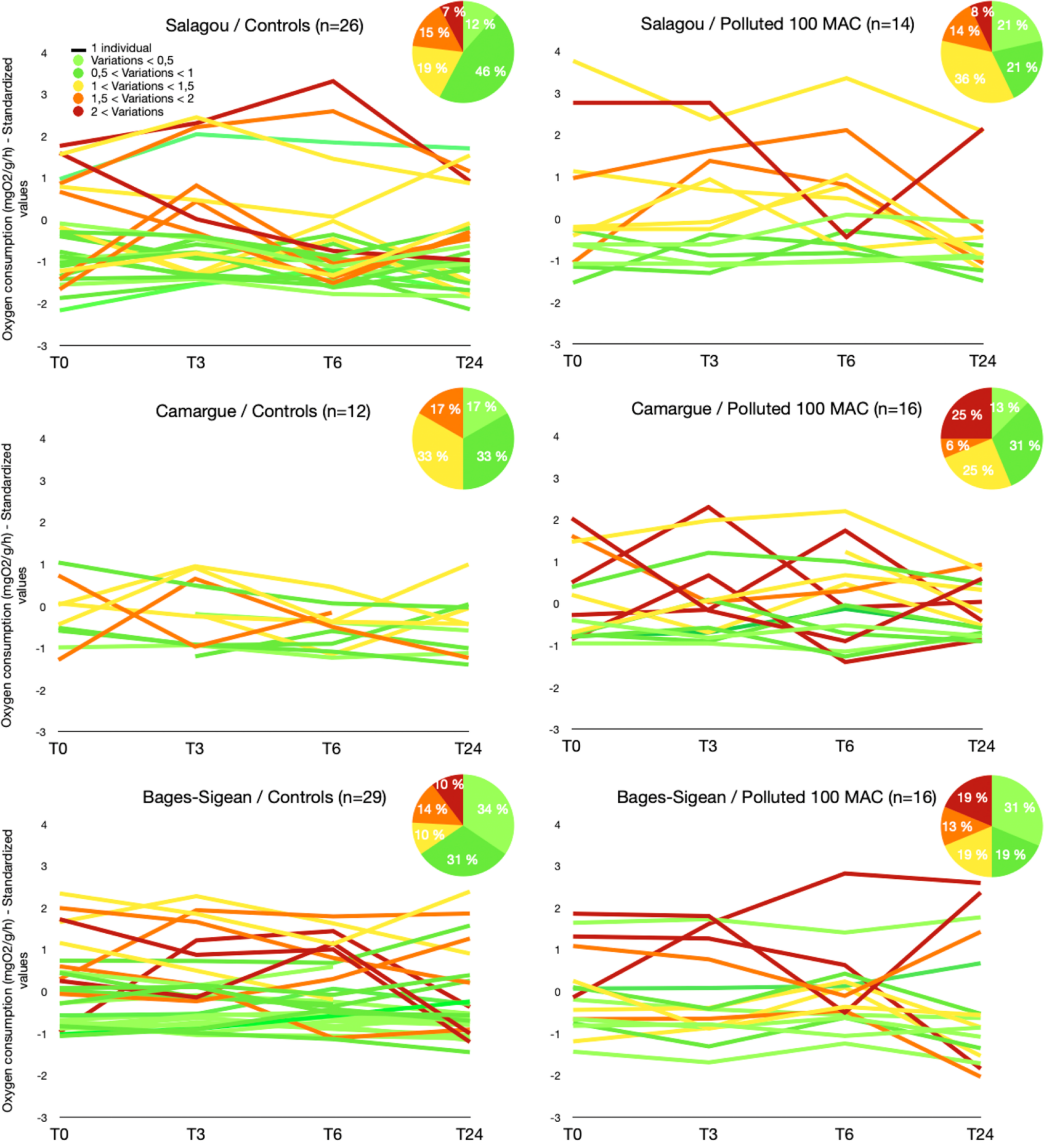
Change with time (T0, T3h, T6h, and T24h) of standardized values (centered and reduced) of dissolved oxygen consumption for each individual (Salagou, Camargue or Bages-Sigean, and controls or polluted 100 MAC). Lines: individuals. Light green line = very stable individual; dark green line = stable individual; yellow line = unstable individual; orange line = agitated individual; dark red line = very agitated individual

All three populations showed a decrease in the percentage of stable individuals (green) when exposed to the pesticide cocktail. This decrease is mainly due to an increase in moderately stable individuals (yellow) for the Salagou population, and it is mainly due to very unstable individuals (red) for the Camargue population. For the Bages-Sigean population, there was an increase in three levels of instability (yellow, orange, and red) with pesticide exposure (Fig. 5).

### Hemolymph osmolarity

**Fig. 6:**
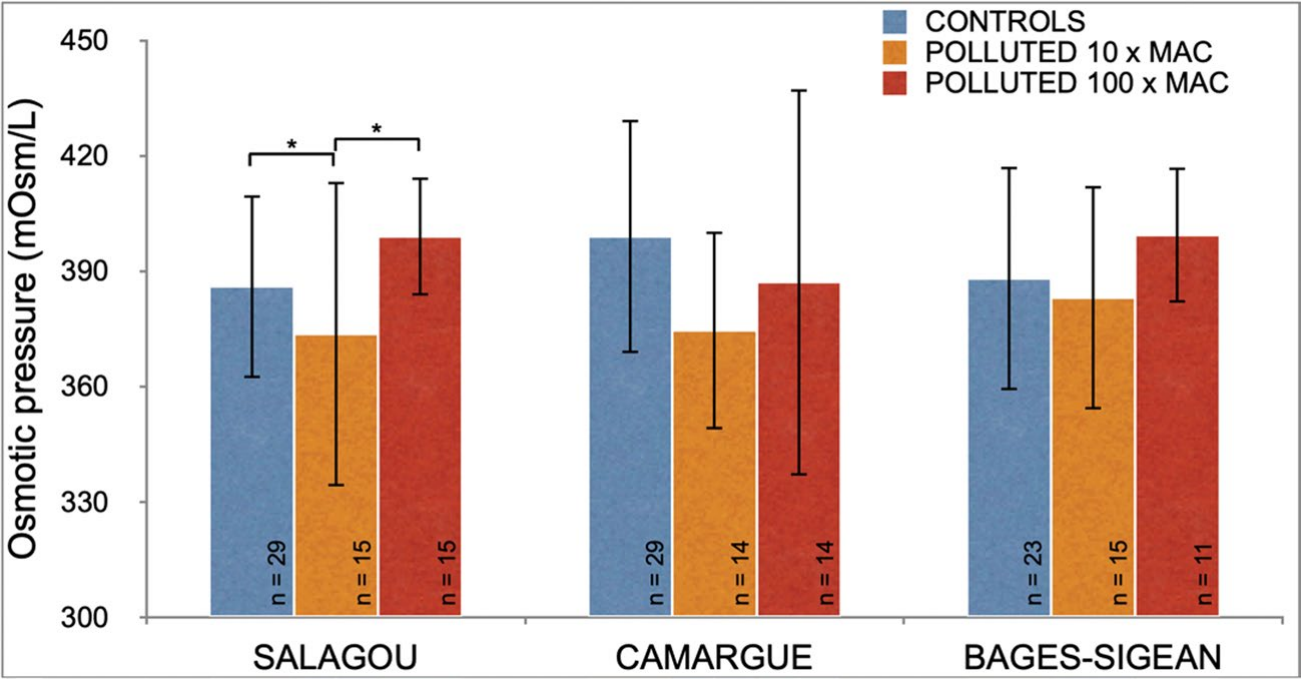
Osmotic pressures (mOsm/L) of three populations of *P. clarkii* hemolymph (Salagou, Camargue or Bages-Sigean) as a function of exposure to a cocktail of oxadiazon/azoxystrobin pollutants (controls in blue, polluted at 10 MAC in orange, polluted at 100 MAC in red). Bar plots with means ± standard deviations

A statistical difference is observed between the different conditions of the Salagou population (*p*-value = 0.008). Individuals exposed to a pollutant concentration of 10 MAC are significantly different from the controls (*p*-value = 0.037) and those exposed to a pollutant concentration of 100 MAC (*p*-value = 0.003). Hemolymph osmotic pressure is decreased after exposure to 10 MAC, but it is similar to the control value when exposed to a concentration of 100 MAC. However, the brackish water populations (Camargue and Bages-Sigean) did not show any difference between the different exposure groups (Fig. 6).

### Activity of digestive enzymes in the hepatopancreas

**Fig. 7:**
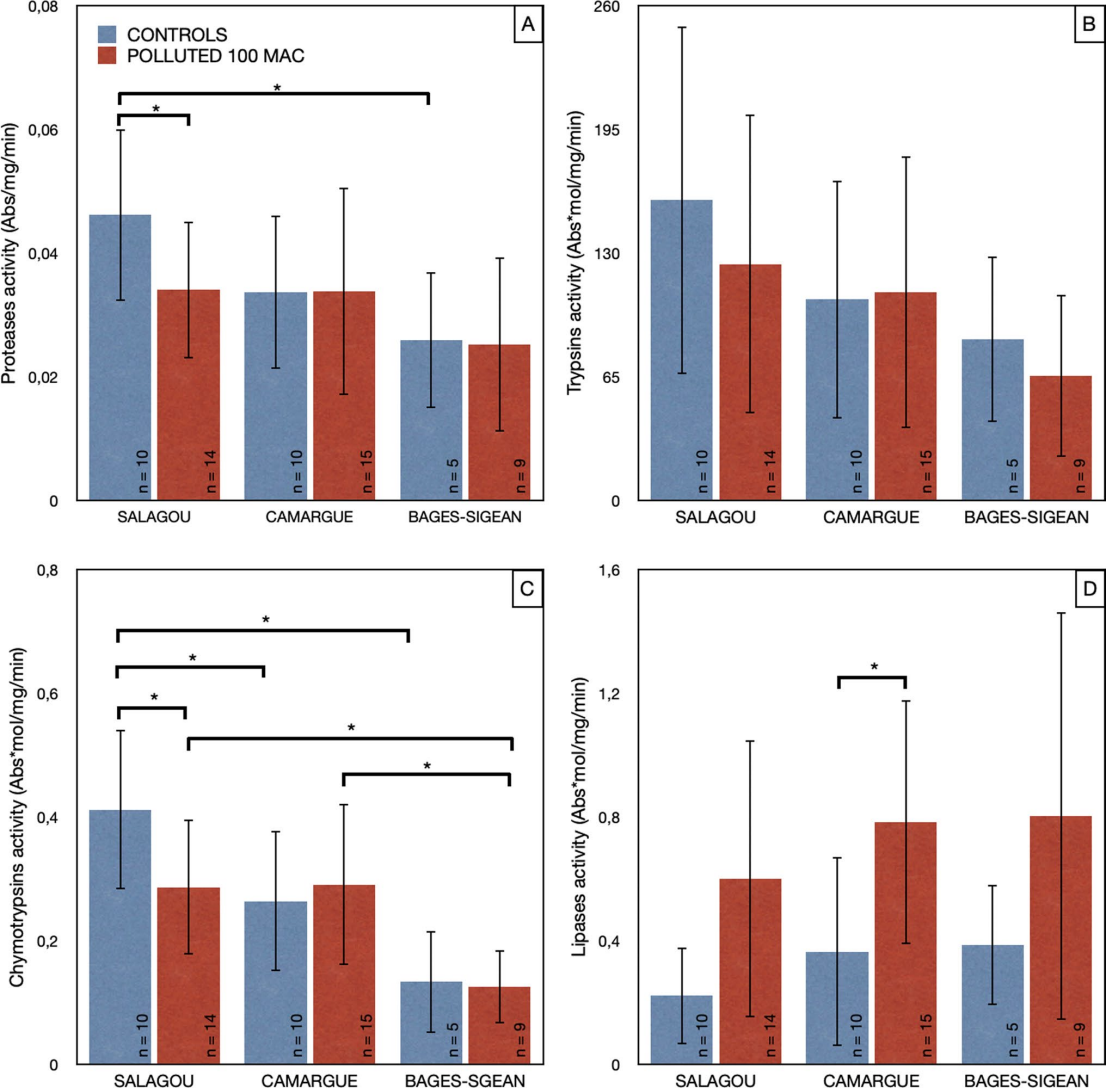
Activity of digestive enzymes in the hepatopancreas of three populations of *P. clarkii* as a function of exposure to a cocktail of oxadiazon/azoxystrobin pollutants (controls in blue, polluted at 100 MAC in red). **A** Protease activity (Abs/mg/min); **B** trypsin activity (Abs*mol/mg/min); **C** chymotrypsin activity (Abs*mol/mg/min); **D** lipase activity (Abs*mol/mg/min). Bar plots with means ± standard deviations

Individuals from the Salagou population have a higher protease activity compared to the Bages-Sigean populations (*p*-value = 0.014). Furthermore, the high activity for Salagou is significantly decreased when individuals are exposed to 100 MAC pollution (*p*-value = 0.003) (Fig. 7A). For trypsin activity, a statistical difference between populations is observed (*p*-value = 0.026): this difference is between Salagou and Bages-Sigean populations (*p*-value = 0.019) (Fig. 7B). For chymotrypsin activity, a statistical difference is observed between the populations (*p*-value = 8E-6). This difference is between Salagou controls and Camargue controls (*p*-value = 0.048) and between Salagou controls and Bages-Sigean controls (*p*-value = 4E-4). The Salagou population presents a decreased chymotrypsin activity when exposed to a 100 MAC pesticide concentration (*p*-value = 0.039). Chymotrypsin activity is also statistically different between exposed individuals: The Bages-Sigean population possesses a significantly lower chymotrypsin activity compared to the Salagou (*p*-value = 0.029) and Camargue (*p*-value = 0.028) populations (Fig. 7C). Also, lipase activity between controls and exposed individuals is significantly different (*p*-value = 4E-4), particularly for individuals of the Camargue population (*p*-value = 0.006) (Fig. 7D).

## Discussion

### Molecular characterization of three populations of crayfish in the South of France

Although the genetic diversity of *P. clarkii* populations in France is generally lower than that of native populations (USA, Mexico) (Oficialdegui et al. 2019), this diversity greatly varies between populations (Almerão et al. 2018). In this study, we report more than 7 different haplotypes in a single population in France, suggesting a higher genetic diversity than expected for the invasive *P. clarkii* crayfish in the South of France. Also, it greatly varies between the three studied populations. The Camargue population has the lowest genetic diversity, 63% of individuals having the H2 haplotype previously described by Almerão et al. (2018) and 37% of them possess the H1 haplotype that has never been reported for populations living in the South of France. In contrast, the Salagou and Bages-Sigean populations showed a much higher genetic diversity, with at least seven different haplotypes. Only one haplotype is shared between Camargue and Bages-Sigean and one between Salagou and Bages-Sigean populations. Therefore, it can be hypothesized a different origin for these populations. The high genetic diversity of the Salagou and Bages-Sigean population may be due to multiple introductions, which are known to favor genetic diversity (Sakaï et al. 2001; Dlugosch and Parker 2008). This phenomenon usually increases the likelihood of introducing genotypes that exhibit high fitness under the ecological conditions found in the new environments (Viard et al. 2016) and, thus, increases the species invasive success. However, we cannot be sure if the low genetic diversity of the Camargue individuals is related to different times of introduction and/or number of introduced individuals, or if it is the result of a loss of genetic diversity due to high environmental stress induced by high pollution and salinity levels in this coastal area. Meineri et al. (2013) indicated that it is very unlikely to find adult red swamp crayfish at a salinity above 10‰ in Camargue area. For juveniles, this threshold drops to 5‰. Therefore, individuals of the Camargue population must avoid high salinities. In 2017, very large inflows of marine water greatly increased the salinity level in the Vaccarès lagoon in the Camargue: In 2016, the salinity varied from 12 to 15‰, whereas in 2019 it varied from 24 to 33‰ (SNPN, 2020). Therefore, it is possible that it led to a high mortality and/or increased migration that acted as a bottleneck, thus reducing genetic diversity.

### Phenotypic variation and its possible link with genetic diversity

#### Can differences in genetic diversity correlate with phenotypic variation?

The study of the energetic balance revealed a difference between populations. The Bages-Sigean population has a higher consumption in dissolved oxygen compared to the other two populations. The habitat around the Bages-Sigean lagoon is more challenging compared to the other two considered in this study, because of a significant and continuous pollution level and high salinity conditions that reach marine values for most of the year (Munaron et al. 2020). Salinity is a key factor in the distribution of aquatic species in their environments (Casellato and Masiero 2011; Dörr et al. 2020; Dobrzycka-Krahel and Fidalgo 2023). Various strategies have been developed by crustaceans (behavioural, ecological, molecular, and physiological mechanisms) to adapt to salinity variations. For example, the bold-type behaviour that is positively correlated with dispersal to the invasion front (Galib et al. 2022) could be even more accentuated when salinity variations occur. In the wetlands around the Bages-Sigean lagoon, crayfish have been proliferating for decades. This environment may have induced the selection of particularly active individuals over a few generations. To support these hypotheses, dedicated behavioral analyses could allow a better characterization of these potential phenotypic differences between populations. Furthermore, the O_2_ consumption for the Camargue population showed a lower inter-individual variability compared to the other two populations. Interestingly, the Camargue population also possesses lower genetic diversity. The low individual variability of oxygen consumption and low genetic diversity suggest local adaptation of the Camargue population. However, in order to determine if there is a link between their energy use and genetic diversity, it would have been necessary to genetically characterize the individuals that were used in the O_2_ consumption analysis (it was not considered in this study).

The energetic balance is not the only physiological parameter that differs between the studied populations. Significantly higher levels of protease activity were recorded for the Salagou individuals compared to the other two populations. As proteases are a non-specific group of enzymes, these differences may be due to different specific enzymes. In this study, only trypsin and chymotrypsin were tested. These among the major digestive endopeptidases of decapods (Tsai et al. 1991; Ma et al. 2005; Vogt *et al.*, 2021). For the Salagou individuals, chymotrypsin activity levels appear significantly higher than those of the other two populations. The Salagou population also shows higher levels of trypsin activity than the Bages-Sigean population. However, no significant difference was observed for lipases. A study focusing on the Chinese crab *Eriocheir sinensis* (H. Milne Edwards, 1853) showed that increases in the activity of digestive enzymes of the hepatopancreas were significantly correlated with decreasing salinity (Wang et al. 2013). The main cause of these variations is related to a higher energy requirement of individuals maintained in freshwater, since they are hyper-osmoregulating. Conversely, in brackish water, individuals approach iso-osmoticity and require less energy from food. In our study on *P. clarkii*, a difference is visible between populations. Indeed, individuals from the Bages-Sigean and Camargue populations, living in brackish water, have a significantly lower chymotrypsin activity compared to individuals from the Salagou population (living only in freshwater). This difference between populations could be due to the adaptation of each population to its environment and the maintenance of an identical level of digestion by individuals regardless of the salinity levels. The more efficient digestion performance of the Salagou population in its own environment (unpolluted freshwater) compared to the other two populations could therefore indicate a local adaptation.

### Metabolic responses to 96-h pesticide exposure

The impact of the pesticide cocktail appears greater for the individuals of the Salagou population which are never exposed to these pollutants in their natural habitat (Aquascop 2016); hence, our hypothesis was confirmed. This population is the only one experiencing a decrease in hemolymphatic osmolarity due to a 10 MAC exposure, although it returns to normal values at higher pollution level (100 MAC). This has already been observed for the fish *Rutilus rutilus* (Linnaeus, 1758) (Katuli et al. 2014) and the shrimp *Penaeus vannamei* (Boone, 1931) (Mena et al. 2020) with exposed individuals having altered hydromineral balance at different levels of exposure to the pesticide dianizon. For the red swamp crayfish, the altered hydromineral balance due to exposure to the azoxystrobin/oxadiazon cocktail at 10 MAC indicates an ion leakage into the environment, whereas, at 100 MAC, an overcompensation occurs with more solutes retained in the body. This could be due to an energy trade-off. When pollution is moderate (10 MAC), the decrease in osmoregulation could be tolerable, individuals allocating a large part of their energy to other functions, such as detoxification. Maintaining the osmoregulatory function is vital, and an imbalance of this key function during an exposition to a stressor can be an indicator of a disturbance in the state of health of the organism (Lignot et al. 2000). For the other two (coastal) populations, the ability to maintain their hydromineral balance even when exposed to the azoxystrobin/oxadiazon cocktail at concentrations of 10 or 100 MAC could be due to a local adaptation process.

Exposed individuals of the Salagou population are also the only ones with a significant decrease in hepatopancreas protease activity compared to the controls. This variation is largely due to a significant decrease in chymotrypsin activity. This suggests that Salagou individuals can either allocate their energy to vital and detoxification functions at the expense of their digestive metabolism (indirect effect of pollution), or that their digestive function is directly targeted (specific pollution effect). This has already been demonstrated in the crab *E. sinensis* after exposure to other herbicides (Yang et al. 2019). Under these conditions, trypsin, amylase, and lipase activities in the hepatopancreas were significantly altered. These changes could be due to cell damage affecting the digestive system, as observed in the lobster *Homarus americanus* (Walker et al. 2010), which can be the reason of the evident lipase activity increase in all the exposure individuals, although only in the Camargue population were statistically different.

For the majority of ecophysiological and ecotoxicological analyses, only averaged responses per experimental group are usually considered. Inter-individual variations such as, for example, those linked to inherently plastic traits, i.e., behavior is rarely taken into account. However, recent studies have emphasized the influence of animal behavior on many ecological aspects using various crustacean species. This can influence growth rates (Biro and Sampson 2015) and also the response to pollution (Steele and Moore 2019) or dispersal (Galib et al. 2022). In this study, when O_2_ consumption rates were recorded, a particularly high degree of agitation was observed among exposed individuals. This can be observed when taking into account the percentage of individuals with very unstable O_2_ consumption as a function of time that increases mean values. This could be explained by an increased exploration behavior of *P. clarkii* exposed to pollution, these individuals possibly trying to escape their stressing environment.

This study highlights the importance of distinguishing between populations in response to environmental changes. Most studies consider that all individuals of a species will have the same survival and dispersal patterns and few integrate intra-specific variations. However, we have shown here that responses to pollution are population specific, which could suggest that local adaptation and plasticity within each population play a key role in the physiological and phenotypic response to environmental conditions. However, further studies integrating antioxidant and detoxification activities, as well as the gene expression of the whole transcriptome, would complement these data and help to elucidate the physiological mechanisms that allow *P. clarkii* to inhabit polluted environments.

## General discussion

Invasive species, including the red swamp crayfish, have demographic and genetic characteristics that make them prone to evolutionary rescue in the event of significant environmental stress (Carlson et al. 2014). Warming water temperatures, increased salinization of freshwater bodies, and increased pollution are all factors that weaken native species while increasing the competitiveness and survival of invasive species such as *P. clarkii* (Dobrzycka-Krahel and Fidalgo 2023). Indeed, abiotic characteristics of the invasion front and habitat alteration are the main drivers explaining the distribution of the red swamp crayfish (Siesa et al. 2011). Therefore, the analysis of rapid adaptive capacities of *P. clarkii* appears particularly relevant to anticipate its future impacts on the environment and biodiversity.

Nevertheless, these adaptations to very specific environments can also be harmful to the persistence of these crayfish populations. When an evolutionary trade-off occurs, adaptation to a particular environmental stress may increase sensitivity to other stressors (Whitehead et al. 2017). Thus, the fitness of a population with a new phenotype may be diminished when it is returned to its ancestral environment. This could be the case for *P. clarkii* individuals inhabiting the Camargue area. Their current phenotype, while adapted to a saltwater environment, may no longer be fully adapted to their ancestral environment (freshwater). Furthermore, evolutionary compromises are often observed in pesticide-resistant populations (Whitehead et al. 2017). This supports the hypothesis that extreme and unpredictable climatic events expected to occur in the coming decades could have various ecological consequences on red swamp crayfish populations depending on their size, genetic variability, and adaptations to specific stresses and environmental conditions in which they are found (Carlson et al. 2014; Whitehead et al. 2017).

Considering the physiological functions analyzed common to all aquatic species (osmoregulatory function, respiration and digestive function), this study gives insights about adaptive and evolutionary mechanisms taking place in invasive species on the invasion front. Due to its global dispersal, tolerance to many abiotic stresses, and biological characteristics (life cycle, rapid reproduction), *P. clarkii* appears as an ideal animal model to conduct research on rapid evolution and local adaptation, in order to identify their processes on a large scale.

## Data Availability

All DNA sequences obtained in this study were submitted to Genbank under the accession numbers (OQ869111, OQ870705, OQ870890, OQ870905-07, OQ870909, OQ870911, OQ871531, OQ871612, OQ872118, OQ872160, OQ872222, OQ915233). And will be a released when the manuscript is accepted.
